# Prediction of Human Activities Based on a New Structure of Skeleton Features and Deep Learning Model

**DOI:** 10.3390/s20174944

**Published:** 2020-09-01

**Authors:** Neziha Jaouedi, Francisco J. Perales, José Maria Buades, Noureddine Boujnah, Med Salim Bouhlel

**Affiliations:** 1 Sciences and Technologies of Image and Telecommunications (SETIT) Laboratory, Sfax 3029, Tunisia; jaouediineziha@gmail.com (N.J.); medsalim.bouhlel@enis.rnu.tn (M.S.B.); 2 Mathematics and Computer Science Department, Universitat de les Illes Balears (UIB), E-07122 Palma, Spain; josemaria.buades@uib.es; 3 Telecommunication Software and Systems Group (TSSG), Waterford Institute of Technology, X91 P20H Waterford, Ireland; bnoureddine@tssg.org

**Keywords:** human activities, action recognition, skeleton features, motion tracking, human detection, deep learning, deep association metric

## Abstract

The recognition of human activities is usually considered to be a simple procedure. Problems occur in complex scenes involving high speeds. Activity prediction using Artificial Intelligence (AI) by numerical analysis has attracted the attention of several researchers. Human activities are an important challenge in various fields. There are many great applications in this area, including smart homes, assistive robotics, human–computer interactions, and improvements in protection in several areas such as security, transport, education, and medicine through the control of falling or aiding in medication consumption for elderly people. The advanced enhancement and success of deep learning techniques in various computer vision applications encourage the use of these methods in video processing. The human presentation is an important challenge in the analysis of human behavior through activity. A person in a video sequence can be described by their motion, skeleton, and/or spatial characteristics. In this paper, we present a novel approach to human activity recognition from videos using the Recurrent Neural Network (RNN) for activity classification and the Convolutional Neural Network (CNN) with a new structure of the human skeleton to carry out feature presentation. The aims of this work are to improve the human presentation through the collection of different features and the exploitation of the new RNN structure for activities. The performance of the proposed approach is evaluated by the RGB-D sensor dataset CAD-60. The experimental results show the performance of the proposed approach through the average error rate obtained (4.5%).

## 1. Introduction

Human activity recognition is a crucial and challenging task in video processing and action classification. Activity recognition is developed in the framework of continuous surveillance of human behavior. It has become the basis for diverse applications, such as healthcare and elderly surveillance, sports injury detection, human position estimation, and home monitoring. Despite the important progress in human activity recognition gathered from video sequences, it remains a delicate problem for many reasons, such as changes in viewpoint and distance from the camera, the complexity of the background, and the diversity of speed.

The extraction of significant features is the most challenging part. Indeed, it influences the performance of the algorithm by reducing the time and complexity of calculations. However, the most traditional methods of human activity recognition [1] are based on handcrafted local features from RGB video taken by 2D cameras that are unable to manage complex activities. Some methods are based on the detection of a moving person by background extraction. Gaussian Background [2], kernel density estimation [3], Visual Background Extractor [4], and Sigma Delta [5] have been used with success in static fonts. Other methods involve motion tracking to characterize human activity. Human tracking is done to control its movement and build trajectories throughout the sequence. Tracking is usually a simple procedure for humans. The problem becomes complex when the speed of the objects is very high. This encourages researchers to develop methods that solve motion tracking problems using computer vision methods such as Optical Flow [6,7], Scale Invariant Feature Transformation (SIFT) [8], Histogram of Oriented Gradient (HOG) [9], and Mean Shift [10]. Since these approaches recognize actions based on the appearance and motion of parts of the human body from RGB video sequence, they lack a 3D structure from the scene.

Therefore, recognizing a human action based only on RGB modality is not enough to overcome current challenges. With the development of artificial intelligence and high computing capacity, some deep learning and transfer learning methods are adopted for the learning and automatic extraction of complex features provided by sensors. The emergence of depth sensor cameras such as Kinect has made activities recognizable by low-cost computer vision methods. These sensors are capable of providing detailed information about human motion, which is more complex for traditional 2D cameras. Indeed, most RGB-D (Depth) cameras currently developed integrate real-time skeleton tracking. The skeleton is a high-level presentation that can be used to describe human activity in a very precise way and is adapted to the challenge of activity analysis and action recognition. However, skeleton data include the coordinates of the human body’s key joints over time. This is an important factor for the motion presentation of each action.

Recently, the recognition of human actions based on the skeleton has attracted the attention of several researchers. Some action recognition approaches using RGB-D cameras and skeleton presentation have been proposed and have an advanced state-of-the-art status. Therefore, the recognition of actions and the analysis of human behaviors in an intelligent home or in an indoor position are becoming more and more important. Indeed, in the field of medicine, there is a rapidly increasing demand for systems to recognize human actions and to quickly detect patients’ physical and mental health problems. Indeed, Gao et al., 2018 [11] developed an application based on human action monitoring for healthcare. This application remotely monitors the status of patients or the elderly. Identifying changes in daily human behavior, such as food preparation, walking, housekeeping, exercise, or sleep allows medical scientists to suggest strategies for diet, exercise, and adherence to treatment. This is particularly important for older people, for whom such systems allow them to live at home longer, in a healthier and safer manner. An equally important component of human activity is the assistive robotic field. David et al., 2019 [12] used the robots to learn human actions by extracting descriptive information about their activity in order to classify them. In this process, activities are integrated into the context of daily living, and normal human activities in indoor positioning are assessed.Based on these methods, we applied three methods in this work: the traditional method for human tracking, skeleton joints, and a deep learning model for human presentation and activity classification. The aim of this work was to propose a new system for human activity analysis based on the presentation of human features in a video sequence. Our contribution is presented in two major stages. In the first stage, we extracted the 2D human skeleton and performed activity classification using the new deep learning model. RGB videos from the CAD-60 dataset were used as input. In the second stage, we performed human activity recognition for continuous streams and real-time videos.

The remainder of this paper is organized as follows. Section 2 describes the development of the human activity techniques and the different presentations of the human descriptor. Section 3 explains our novel proposed approach, and the last section presents the experimental results and summarizes the comparison with the state-of-the-art methods.

## 2. Related Works

The recognition of human activities using intelligent techniques is usually based on two major stages: feature extraction and action classification. Features are the most important information to describe human activity; they can be visual, such as pixel intensity, texture, or temporal information such as the motion direction or trajectory path. To obtain an efficient vector of human activity information, many researchers have used spatial or visual features. AlbuSlava 2016 [13] and Majed Latah 2017 [14] proposed a new method based only on spatial and contextual features to predict customer behavior and human actions. Indeed, they exploited the performance of the Convolutional Neural Network (CNN) and 3D-CNN to extract spatial information for emotions and action classification. Other authors demonstrated that human actions can be better described using motion and speed. Murad and Ryun 2017 [15] and Qin et al. [16] applied body-worn sensors and Long Short Term Memory (LSTM) Recurrent Neural Networks for human motion description. The latter involves gyroscope and accelerometer measures. In the same context, Ning et al., 2017 [17] only used temporal features based on the local optical flow of the global human silhouette for human action recognition. Despite the level of performance found, these methods remain sensitive in complex scenes, which present variations in background, scale, and texture. These make the recognition of human activities difficult.

To enhance the results of human action classification, researchers in this field proposed a combination of spatial and temporal features. Here, Nicolas et al., 2016 [18] applied a novel model to learn spatio-temporal features using Gated Recurrent Units (GRU) with Recurrent Convolution Networks (RCN). This model is based on the pre-trained VGG-16 on an ImageNet transfer learning Model. Xu et al., 2016 [19] and Baldominos et al. [20] proposed the Recurrent Convolution Neural Networks (RCNN) model for human action recognition using GoogleLeNet architecture. Here, the authors exploited the advantages of CNN and RNN to extract the temporal and spatial features. Then, they used the GoogleLeNet architecture to combine features and compute the accuracy of video classification. In the same context, Zhang et al., 2016 [21] presented deep-learned spatio-temporal features using a vector of locally aggregated descriptors to detect motion descriptor and SIFT geometric information to predict motion descriptors. An Independent Subspace Analysis (ISA) was used for contextual feature extraction. Zhao et al., 2017 [22] used other techniques to extract human features. They combined the RNN model with GRU hidden units and 3D-CNN architectures. These techniques were used successfully to determine the spatio-temporal features of some human actions that do not require the complete presentation of the human body.

Recently, there has been a growing interest on depth information and the skeleton presentation of human activities. Faria et al., 2012 [23] applied a dynamic Bayesian mixture model for human activity prediction based on skeleton features. This model is designed to combine multiple classifier probabilities into a single form by assigning weights to counterbalance the probabilities as posterior probabilities. Koppula et al., 2013 [24] modeled the skeleton of a human being using Hidden Markov Models (HMM). Nodes present the objects and activities, and the edges present the relationships between objects and activities. For the classification of the latter, [24] used the Support Vector Machines (SVM) method. Bingbing et al., 2013 [25] proposed a novel feature extraction technique using the fusion of grayscale and depth frame to extract 3D spatial and temporal descriptors of human activities.

Indeed, depth filters are used to remove false grayscale human detection. Wang et al., 2014 [26] proposed a new feature based on a 3D skeleton and Local Occupation Model (LOM) for learning human actions. The objective of the LOM is to reduce the use of 3D human joints. In fact, this study defined each action by the human’s movement joints; for example, for a person drinking water, only the joint of the hand would be extracted. To address the same aim, Shan and Akella 2014 [27] and Enea et al., 2016 [28] used RGB-D sensors for human skeleton detection and kinetic energy to identify the key poses that present intense positions of action in a large space. The 3D presentation of human joints is exploited by the SVM technique to predict and determine human actions. Gaglio et al., 2015 [29] combined three machine learning techniques to predict human activities. They used the K-means method to detect a human 3D skeleton posture, the SVM method for classification, and the Hidden Markov Models (HMM) to model activity.

More recently, Manzi et al., 2017 [30] applied RGB-D sensors to select human skeleton features, the K-means method for posture selection, and Sequential Minimal Optimization for training data. The purpose of this architecture was to select and demonstrate that the minimum number of key poses is sufficient to describe and recognize a human activity. Srijanet al., 2018 [31], Cruz et al. [32], and Khaire et al. [33] proposed and developed a method based on the combination of skeleton and contextual feature extraction. The skeleton features were extracted by the RGB-D sensor and the CNN and LSTM models. The contextual features were detected using the CNN model. In addition, Yanliet al., 2018 [34] proposed a View-guided Skeleton-CNN (VS-CNN) model for human arbitrary view and human action recognition which carries on weakening view differences by visualizing skeleton sequences and covers a larger range of view angles. Hug et al., 2019 [35] applied an action recognition model based on the transformation of the skeleton to a spatial presentation using the conversion of the distance values of two joints to color points, and they used the DenseNet CNN model for action classification. We finish this section by presenting the major conclusions extracted by the two surveys of Wan et al., 2018 [36] and Pham et al., 2019 [37]. These authors found that methods based on human pose estimation and skeleton feature extraction can achieve higher classification rates.

Based on this finding, we developed the contribution presented in this paper. We present a new model that can be used for continuous and online human activity recognition using pose estimation features. Our new model can exploit different types of features: temporal using the Kalman filter, visual using the CNN model Inception V3, and 2D skeleton features using the pose estimation technique. All of these features are used as inputs of our GRU RNN model for online human activity prediction. Table 1 presents a summary of different methods and our proposed technique.

## 3. Proposed System for the Classification of Human Activities

**Figure 1:**
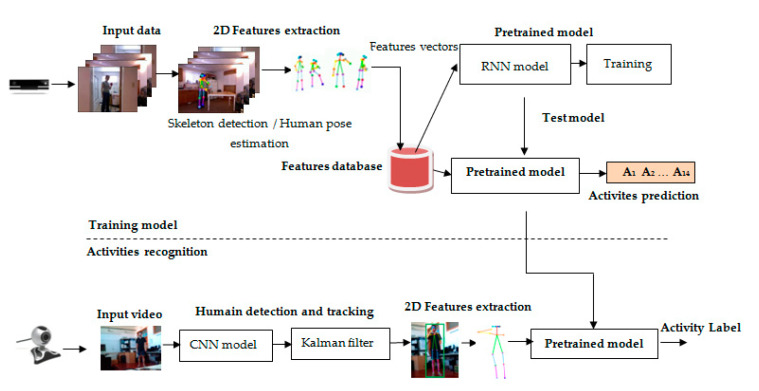
The newly proposed model for human activity recognition. Our model is divided into two parts: model training and activity recognition. This model is based on human pose estimation and human tracking.

Our system for the analysis of human activities relies on the extraction of relevant features from human presentation video sequences. Our system consists of two parts: the first part addresses the construction of a new classification model based on the 2D human skeleton. The performance of this model was evaluated using inside-home activities from the CAD-60 database (Section 3.1). The second part proposes continuous and real-time human activity recognition. In this part, three types of features are exploited to present human activities: visual, temporal, and 2D human skeleton. Figure 1 presents an overview of our proposed system for activity classification and action recognition.

### 3.1. Model Training

In this section, we explain the steps involved in training the classification model. This is based on two major stages: the first is the extraction of 2D features using skeleton detection and human pose estimation; the second is the pre-training of a new deep learning model based on activity classification.

#### 3.1.1. Human Pose Estimation and Skeleton Detection

One of the major problems with human activity recognition is determining how the human pose can be described and estimated. Human pose estimation presents the position and orientation of a person using the detection of their skeleton. This includes the joints or key points of the human body—for instance, the elbows and knees. The position and orientation of each joint affect the position and orientation of the next joint in the skeleton. The challenge of human pose estimation is defined as the most accurate computer vision techniques that predict the locations of human key points such as the nose, elbow, wrist, shoulder, hip, and ankle.

**Figure 2:**
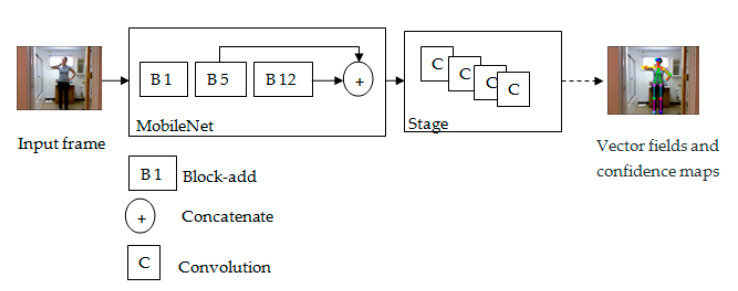
The MobileNet architecture for skeleton joint representation.

**Figure 3:**
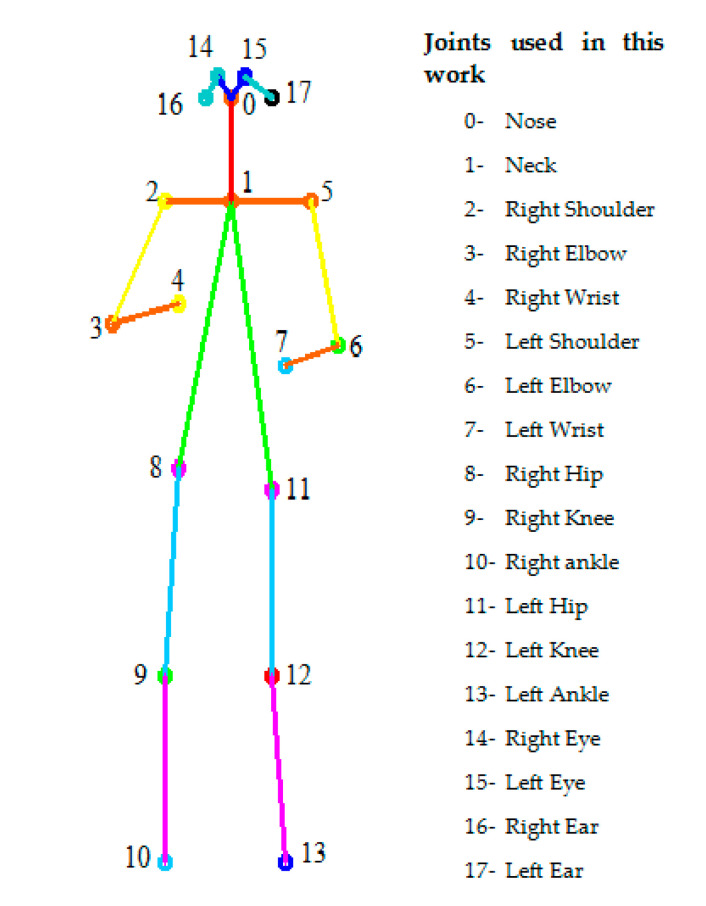
The human skeleton presentation used in our paper. This skeleton model presents 18 joints in which each joint is projected in the 2D plane. For the collection of human features, we used 36 values (18 × 2), for example, nose_x, nose_y, neck_x, neck_y, Right_shoulder_x, Right_shoulder_y, Right_elbow_x, Right_elbow_y, etc.

In our study, we used the COCO (Common Object in Context, http://cocodataset.org/#keypoints-eval) representation with the MobileNet CNN model for key point detection and 2D skeleton reconstruction rather than using the skeleton information obtained from the Kinect tool. The MobileNet model takes video frames as input and concatenates the outputs of Block-add 5 and Block-add 12. The information collected from these blocks is used as input of the first stage to present possible locations of human key points and confidence maps. The MobileNet structure used in this paper for skeleton presentation contains two stages. The second stage takes the concatenation of the previous stage outputs to present the total human key points and the confidence maps. Figure 2 depicts our MobileNet structure for skeleton detection. The MobileNet model collects 18 joints from the human body thanks to its two layers—depthwise convolution and pointwise convolution—in its blocks. MobileNet is trained using the COCO model presented in Figure 3. Each joint is presented using 2D coordinates (x, y); for example, the joint of the nose is defined by (Nose_x, Nose_y). The computation of the joints’ locations using the COCO model is based on Object Key point Similarity (OKS). The OKS technique focuses on the Euclidean distance between each corresponding ground truth normalized by the scale of the person and detected key points and visibility flags. The computation of OKS values is based on an un-normalized Gaussian function with a given standard deviation. The total number of selection features is double the number of joints.

The selected human features are presented in a CSV file containing 36 values from 18 joints. The feature vectors computed are concatenated to build features of a dataset matrix associated with human activities in a video. Human activity features are presented as matrices with 37 columns by N rows; the columns present 36 values of skeleton joints and classification of the skeleton presentation. The rows correspond to features from different frames of human activities in the Cornell Activity Dataset (CAD )60 dataset.

#### 3.1.2. Deep Learning Model

Recurrent neural networks are able to learn long-term features and dependencies from sequential and temporal data. RNNs have a stack of nonlinear units where at least one connection between units forms a directed cycle. A well-trained RNN can model any dynamic system. However, RNN training is mainly affected by long-term dependency learning. RNN units can handle large datasets using deep architectures. RNN can contain two unit types: LSTM and GRU.

The LSTM has been developed to address gradient explosion problems and can be considered as a deep neural network architecture once unwound over time. The main component of the LSTM layer is a unit called a memory block. An LSTM block has three gates: input, output, and forget gates. These gates can be considered to involve operations of writing, reading, and resetting the cells. An LSTM cell state is the key component that carries information between LSTM blocks.

A Gated Recurrent Unit (GRU) is part of a specific recurrent neural network model that intends to use connections through a sequence of nodes to perform memory and clustering-related automatic learning tasks. GRUs help adjust the input weights of the neural networks to solve the endangered gradient problem, which is a common problem in recurrent neural networks. The GRUs have what is called an update gate and a reset gate. Using these two gates, the model refines the results by controlling the flow of information across the model. Similar to other types of recurring network models, models with gated recurring units can retain information over a period of time, so one of the simplest ways to describe these types of technologies is to use a network of neurons of type “centered on the memory”. For this reason, in this work, we chose to use the RNN with GRUs.

### 3.2. Activity Recognition

To improve the performance of our human activity recognition system, we developed four tasks, which are presented in this section, as follows:

- The collection of 14 activities in one input video and real-time reading of human activities using a standard webcam.
- Human tracking using the pre-trained transfer learning Convolutional Neural Network (CNN) model Inception V3 and the Kalman filter.
- Two-dimensional feature extraction using human pose estimation and skeleton detection.
- Human activity recognition.

**Figure 4:**
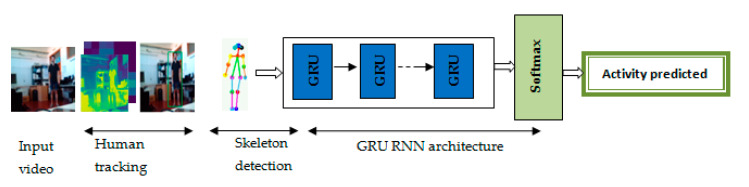
The structure of our human activity recognition system.

The classification and recognition of human activities have become important tasks for many applications in the fields of medicine, smart surveillance, and video games. In this study, we developed a new activity recognition system, which is presented in Figure 4. The input of our system is a continuous video that groups 14 human activities involving one or many people. Each activity is presented as a short video of 3 s. To properly recognize human activities throughout the frames of each mini video, we used Inception V3 to extract the visual characteristics of a person in each frame. Moreover, to locate the moving person in a video sequence, we used a linear Kalman filter [38].

Inception V3 is a transfer learning model, and it has a deep architecture. It consists of symmetrical and asymmetric basic components, including convolutional layers, maximum and average clustering layers, concatenation layers, dropped layers, and fully connected layers. The layer of batch normalization is widely used in this model. The system loss is calculated via Softmax. To build our model, video sequences presented by these frames are used as the input. These are used by the Inception V3 model to extract the output of the last average pooling layer that presents the characteristics framework. Inception V3 seemed to be the best transfer learning model compromise between resource consumption, learning time, and performance. Moreover, the concatenation layer of convolutional and pooling layers makes the features more specific.

**Figure 5:**
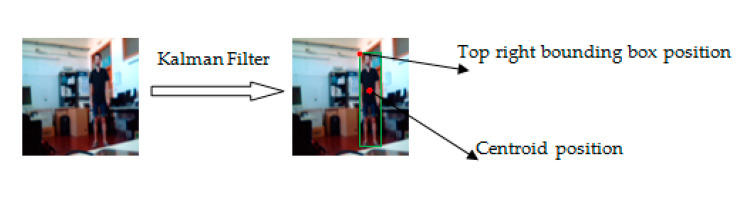
Human tracking using the Kalman filter.

Therefore, the tracking of a moving person consists of locating their position in a video sequence for each time point. The tracking algorithms’ average shift and edge detection play important roles in computer vision. The Kalman filter is based on a state-space approach to estimate the state of the system using the distribution function of the Kalman filter. This approach can recover lost traces, making them the most useful tracking algorithms. The Kalman filter can solve tracking problems based on the state space and measurement equations. The two steps used to track a person through the Kalman filter are prediction and correction. During the prediction step, the current time state is estimated from the previous state. This estimation is also called a priori state estimation. It does not require a measurement value. The Kalman filter estimates the position, speed, and acceleration of the person in each frame of the video. It can track by using the linear model and Gaussian noise to obtain better results with a minimal mean square error. It is a recursive estimator, which means that to estimate the current state, the previous state and its current measurements are required. These two are enough to assess the current state. The Kalman filter averages the prediction of the state of the system with a new measurement using a weighted average phenomenon to obtain more precise estimated values. The goal of human tracking in this part of our system is to link the frames, because we need to visualize and predict human activity in each frame. In this stage, human activity recognition in a video using the linear Kalman filter has the most critical role. It can extract the bounding box using a centroid position and track the moving person in a video sequence (Figure 5). This step can reduce the computing time and improve the performance of our model. The sequential use of Inception V3 and the Kalman filter is considered an essential step in our recognition system. The next step in our system is skeleton extraction and human activity classification.

## 4. Experimental Results

### 4.1. Dataset

**Figure 6:**
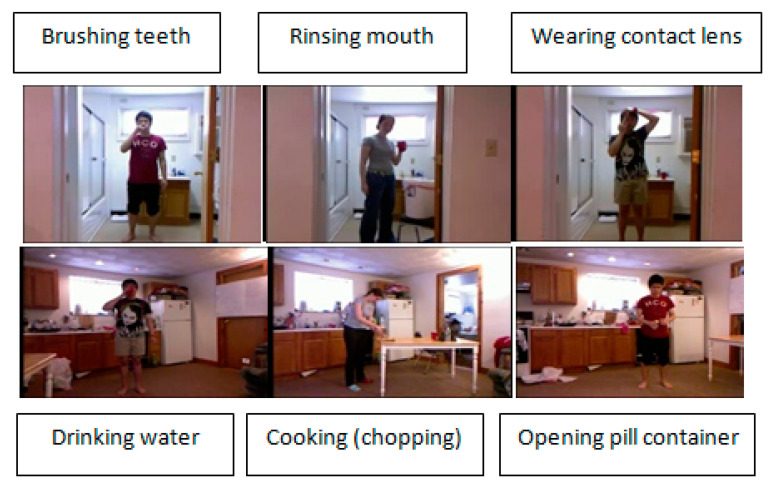
Six activity classes included in the CAD-60 dataset. The video samples were captured by Microsoft Kinect sensors concurrently at 25 fps. The activities were performed in the bathroom and kitchen.

The Cornell Activity Dataset CAD-60 includes RGB-D activity sequences for human indoor activities. These activities are acquired using an RGB-D sensor at a rate of 25 frames per second. CAD-60 contains information on RGB images, depth, and source code that can be used to detect skeletal coordinates of 15 human joints. This activity was presented by Sung et al., 2012 [39]. However, according to the description given by [39], this database presents 60 video sequences of five people. The version published for the system of recognition of the human activities presents 56 video sequences grouped into 14 classes: brushing teeth, cooking (chopping), cooking (stirring), water drinking, opening a pill container, random, relaxing on the couch, talking on the phone, wearing contact lenses, working on a computer, and writing on the whiteboard. These activities are done by four people (two boys and two girls) in two different places: the bathroom and the kitchen. To visualize the human activities of the CAD-60 database, we present some of them in Figure 6.

### 4.2. Implementation Details

**Figure 7:**
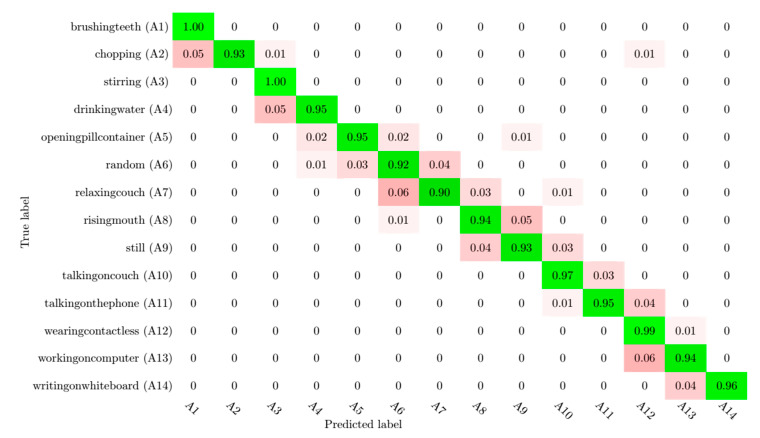
Confusion matrix of the proposed human activity recognition system. The true labels are presented in the rows, and the labels predicted by the proposed model are presented in the columns.

Python, with the support of the Keras framework using TensorFlow, was installed on a laptop computer with the Intel Core i7-8550U 8th Generation Processor 4.0 GHz and 8 GB of memory. In the first part of the evaluation of our system, the skeleton data were collected from the RGB videos of the CAD-60 dataset. The skeleton presentation considered 18 joints. Initially, the human skeleton presented in CAD-60 dataset, which was extracted using the Kinect sensor, had only 15 joints. This presentation did not include the eyes and ears. However, a central joint between the hip and the shoulder was added. The features selected from the experimental dataset included 36 values, and the size of the feature database and the CSV file was around 80,300 values. After the detection of 2D human features using human pose estimation and skeleton detection, we split the dataset of features obtained into one dataset with three-quarters of the features for training and a dataset with one-quarter of the features for the test. The collection of RGB features test frames was used for activity recognition. For activity classification, the RNN model was trained for 60 epochs with a batch size of 100, and the weights were adjusted by the Adam optimizer [40] used with default parameters (0.0001). Additionally, we used the categorical cross-entropy loss technique and the accuracy metrics technique. The best recognition achieved 100%. The recognition rate of each activity class of CAD-60 is provided in Figure 7 via a confusion matrix. Moreover, Table 1 illustrates the different recall, precision, and F1 metrics values of the 14 activities, in which A1, A2, …, A14 correspond respectively to brushing teeth, chopping, …, writing on the whiteboard. The latter presents high values. All actions present values of precision and recall higher than 90%; for example, the recall values for brushing teeth and stirring actions were found to be 100%. Additionally, the recall value for wearing contact lenses was 99%. The latter can be changed according to the human’s location. Table 2 presents the distribution of recall values in different actions.

In this study, our system was used to evaluate indoor activities and carry out action recognition using the CAD-60 in five locations. The purpose of this was to eliminate complex scenes with moving backgrounds. The classification time of the proposed method did not exceed 30 min for training and testing combined, but the time taken for feature collection and CSV file construction of the CAD 60 was more than one day. Our model maintained its level of performance in the different locations, as presented in Table 3.

### 4.3. Activity Recognition

**Figure 8:**
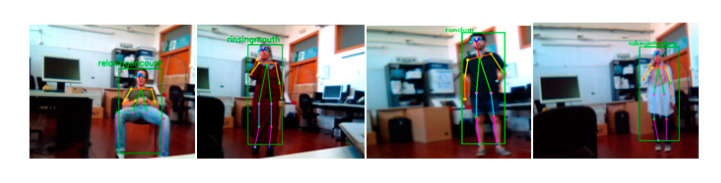
Some activities classified for four people in the UIB lab: relaxing on the couch, rinsing teeth, random, and talking on the phone.

The second part of our system is devoted to the recognition of human activities. We considered the video containing the collection frames of the skeleton information in the test database to be the input and the predicted activity to be the output. To make our system more efficient, we evaluated it on a real-time basis. The proposed system was tested through a collaboration with students from the UIB (Universitat de les Illes Balears) laboratory, where each person performed one of 14 activities presented in the CAD-60. Each activity lasted 3 s to allow our system to better predict the movements that occurred. Real-time human activity recognition is different from continuous human activity recognition. Indeed, the performance of the first system could not be measured, but we were able to observe the results. In the experimental results, in 3 s of human activity (90 frames), incorrect activity predictions were also observed. Some of the real-time results of activity prediction are presented in Figure 8, including the detection and tracking of a moving person using a bounding box. Furthermore, human pose estimation was performed using the detection of human joints and skeleton presentation. The results show that human tracking in real-time systems is an important step for detecting and tracking people. Despite the difference between the background of the CAD-60 dataset and our scene (Laboratory of UIB University Figure 8), the average accuracy rate was around 96%.

### 4.4. Comparisons with the State-Of-The-Art Method

A comparison of the state-of-the-art method using the CAD-60 dataset with our system is summarized in Table 4. We compared the proposed system with similar models that were used between 2014 and 2019. Fariaet al., 2018 [20] proposed an approach based on the dynamic Bayesian mixture model for the skeleton presentation of human activities using a single classifier, and the average accuracy of the proposed approach was 91.9%. In 2015, Gaglio et al. [29] proposed a method based on 3D postural data for feature extraction and Support Vector Machine with the Hidden Markov Model for activity classification. The accuracy of this method reached 77.3%. Recently, Cippitelli et al. [28] in 2016 and David et al. [12] in 2018 developed new approaches for activity classification using RGBD sensors and Support Vector Machine. The average activity accuracy levels obtained were, respectively, 93.5% and 92.3%. In 2019, Shabaninia et al. [41] considered weighted 3D joints for human activity presentation. The average accuracy level obtained was 94.94%.

Moreover, we can compare our system with recent work carried in 2019 with different datasets. Indeed, Jan et al. [42] developed a new approach for motion capture based on skeleton presentation and deep convolutional networks. The performance of this approach was evaluated using the HDM05-122 dataset, and the accuracy rate obtained was 91.5%. Kai et al. [43] proposed a new model for human action recognition based on the Convolutional LSTM Network using multi-neighborhood graph convolution and multiple LSTMs. The model was tested with the Nanyang Technological University (NTU)dataset, and an accuracy level of 93.8% was achieved. In the same context, Petr et al. [44] and Fabio et al. [45] developed an action recognition system based on the bidirectional LSTM network to analyze the accuracy gap in the expressive power of 2D and 3D skeletons using the PKU-MMD and HDM05-15 datasets. The accuracy rates obtained were 92.2% and 88.88%.

## 5. Conclusions and Future Work

In this paper, we presented an effective system for human activity classification and action recognition based on the following steps: training of a new deep learning model using human skeleton features and activity recognition using the CNN model, Kalman filter, and the trained model. In the first step, we exploited the pose estimation technique to extract the human skeleton presentation and the movement of joints for the extraction of human features. The latter were used by a new deep learning model based on RNN with GRU. The better exploitation of the deep learning model RNN with key point features improved the accuracy of human activity classification. In the second step, we evaluated our system using a continuous video sequence (collective action from the CAD-60 dataset and real-time human actions). In this step, we added two techniques for human tracking in each frame into our system. We used Inception V3 followed by Kalman filtering to enhance human motion detection and tracking. Our system achieved great performance for activity classification. A hybrid combination of the transfer learning CNN model, human tracking, skeleton features, and the deep learning RNN model with Gated Recurrent Unit improved the cognitive capability of the system. A comparative study with existing approaches in related works was carried out. This comparison led to the conclusion that the proposed system gives satisfactory results.

As future work, we plan to develop assistive robot applications to help people with social aspects. Indeed, assistive robots could be equipped with the ability to learn human activities. These robots are an active research topic in which various solutions are being developed to improve the life quality of aging people and children. For example, a robot could help children on the autism spectrum connect with other children and it could help aging people to perform daily tasks, as well as providing healthcare assistance. Our application combines telecommunication infrastructure, human activities, and deep learning architectures.

In this work we focus to develop new system for human activity analysis inside of home. Our system based on the uses of skeleton data. We exploited in this paper two types of deep learning models: Convolutional Neural Network and Recurrent Neural Network with the Kalman filter for human tracking in real time. Our system is evaluated by CAD 60 dataset. According to the experimental results and the comparison with the state of the arts we can conclude that our system has a good performance.

## Figures and Tables

**Table 1:** State-of-the-art methods and their interpretation.

Authors	Methods	Interpretation
AlbuSlava 2016 [13] and Majed Latah 2017 [14]	3D CNN	Spatial features
Murad and Ryun 2017 [15] and Qin et al. [16]	Deep recurrent neural networks and multimodal sensors	Motion features
Ning et al., 2017 [17]	Local optical flow of a global human silhouette	Motion features
Nicolas et al., 2016 [18]	GRU + RCN	Spatio-temporal features
Xu et al., 2016 [19] and Baldominos et al. [20]	RCNN	Spatio-temporal features
Zhang et al., 2016 [21]	Vector of locally aggregated descriptors, SIFT and ISA	Spatio-temporal features
Zhao et al., 2017 [22]	RNN + GRU + 3D CNN	Spatio-temporal features
Faria et al., 2012 [23]	Dynamic Bayesian mixture model	Skeleton features
Koppula et al., 2013 [24]	HMM	Skeleton features
Bingbing et al., 2013 [25]	Histogram of oriented gradient and SVM	Spatio-temporal features
Wang et al., 2014 [26]	LOM	Skeleton features
Shan and Akella 2014 [27] and Enea et al., 2016 [28]	Pose Kinetic Energy + SVM	Skeleton features
Gaglio et al., 2015 [29]	Kmeans + HMM + SVM	Skeleton features
Manzi et al.,2017 [30]	Kmeans + Sequential Minimal Optimization	Skeleton features
Srijan et al., 2018 [31], Cruz et al. [32] and Khaire et al. [33]	RGB-D + CNN + LSTM model	Skeleton and contextual features
Yanli et al., 2018 [34]	VS-CNN	Skeleton and contextual features
Hug et al., 2019 [35]	The conversion of the distance value of two joints to colors points + CNN	Skeleton and contextual features
**Proposed approach**	**CNN (Inception V3 + mobileNet) + GRU + RNN + Kalman filter**	**Skeleton + spatio-temporal features**

CNN: Convolutional Neural Network, GRU: Gated Recurrent Units, LOM: Local Occupation Model, LSTM: Long Short Term Memory, RCN: Recurrent Convolution Networks, RNN: Recurrent Neural Network, SVM: Support Vector Machines, VS-CNN: View-guided Skeleton-CNN.

**Table 2:** Classification recall, precision, and F1 of the Cornell Activity Dataset (CAD) 60 dataset.

	A1	A2	A3	A4	A5	A6	A7	A8	A9	A10	A11	A12	A13	A14
**Recall**	1	0.93	1	0.95	0.95	0.92	0.90	0.94	0.93	0.97	0.95	0.99	0.94	0.96
**Precision**	0.95	1	0.94	0.96	0.96	0.91	0.95	0.93	0.93	0.95	0.97	0.90	0.95	1
**F1**	0.97	0.96	0.96	0.95	0.95	0.91	0.92	0.93	0.93	0.96	0.96	0.94	0.94	0.98

**Table 3:** Performance of our proposed system according to human locations using the CAD-60. Five locations are presented: bathroom, bedroom, kitchen, living room, and office.

Location	Activity	Prediction (%)
Bathroom	Brushing teeth Rinsing mouth Wearing contact lenses	100% 94% 99%
Bedroom	Drinking water Opening pill container	95% 95%
Kitchen	Cooking (chopping) Cooking (stirring) Still	93% 100% 93%
Living room	Random Relaxing on couch Talking on phone Talking on couch	92% 90% 95% 97%
Office	Writing on board Working on computer	96% 94%
Average		**95.5%**

**Table 4:** Average recognition accuracies (%) of our approach and comparison with previous works using the CAD-60 dataset. The best accuracy level is presented in bold.

Methods	Year	Acc. (%)
Dynamic Bayesian Mixture Model [23]	2014	91.9%
Support Vector Machine + Hidden Markov Model [26]	2015	77.3%
Multiclass Support Vector Machine [25]	2016	93.5
Classifier Ensemble [12]	2018	92.3%
Weighted 3D joints [41]	2019	94.4%
Our System	2020	**95.5%**
